# The Contact Allergen NiSO_4_ Triggers a Distinct Molecular Response in Primary Human Dendritic Cells Compared to Bacterial LPS

**DOI:** 10.3389/fimmu.2021.644700

**Published:** 2021-03-11

**Authors:** Tessa Höper, Katherina Siewert, Verónica I. Dumit, Martin von Bergen, Kristin Schubert, Andrea Haase

**Affiliations:** ^1^Department of Chemical and Product Safety, German Federal Institute for Risk Assessment (BfR), Berlin, Germany; ^2^Department of Clinical Pharmacy and Biochemistry, Institute of Pharmacy, Freie Universität Berlin, Berlin, Germany; ^3^Department of Molecular Systems Biology, Helmholtz-Centre for Environmental Research - UFZ, Leipzig, Germany; ^4^Institute of Biochemistry, Leipzig University, Leipzig, Germany

**Keywords:** monocyte-derived dendritic cells, allergic contact dermatitis, proteomics, nickel, LPS

## Abstract

Dendritic cells (DC) play a central role in the pathogenesis of allergic contact dermatitis (ACD), the most prevalent form of immunotoxicity in humans. However, knowledge on allergy-induced DC maturation is still limited and proteomic studies, allowing to unravel molecular effects of allergens, remain scarce. Therefore, we conducted a global proteomic analysis of human monocyte-derived dendritic cells (MoDC) treated with NiSO_4_, the most prominent cause of ACD and compared proteomic alterations induced by NiSO_4_ to the bacterial trigger lipopolysaccharide (LPS). Both substances possess a similar toll-like receptor (TLR) 4 binding capacity, allowing to identify allergy-specific effects compared to bacterial activation. MoDCs treated for 24 h with 2.5 μg/ml LPS displayed a robust immunological response, characterized by upregulation of DC activation markers, secretion of pro-inflammatory cytokines and stimulation of T cell proliferation. Similar immunological reactions were observed after treatment with 400 μM NiSO_4_ but less pronounced. Both substances triggered TLR4 and triggering receptor expressed on myeloid cells (TREM) 1 signaling. However, NiSO_4_ also activated hypoxic and apoptotic pathways, which might have overshadowed initial signaling. Moreover, our proteomic data support the importance of nuclear factor erythroid 2-related factor 2 (Nrf2) as a key player in sensitization since many Nrf2 targets genes were strongly upregulated on protein and gene level selectively after treatment with NiSO_4_. Strikingly, NiSO_4_ stimulation induced cellular cholesterol depletion which was counteracted by the induction of genes and proteins relevant for cholesterol biosynthesis. Our proteomic study allowed for the first time to better characterize some of the fundamental differences between NiSO_4_ and LPS-triggered activation of MoDCs, providing an essential contribution to the molecular understanding of contact allergy.

## Introduction

Dendritic cells (DC) play a crucial role during the immune response given their linking function between innate and adaptive immunity. Immature DCs are phagocytic and sample antigens from surrounding tissues. The presence of additional stimuli, for instance activation via pattern recognition receptors such as toll-like receptors (TLR), triggers maturation of DCs. Upon maturation, the cells will partially lose their phagocytic characteristics and start migrating to draining lymph nodes while they undergo metabolic and phenotypic alterations (1–3) facilitating the presentation of the captured antigen to prime naïve T cells. These alterations include the secretion of different cytokines [e g., interleukin (IL) 1β, IL-6, IL-12 or tumor necrosis factor (TNF) α and the upregulation of antigen-presenting molecules and other DC surface proteins such as adhesion molecules, chemokine receptors, and co-stimulatory proteins. Particularly well described is the upregulation of the cluster of differentiation (CD) 86, CD54, CD40 and CD83 (4). A metabolic shift toward glycolysis supplies sufficient energy for the maturation of activated DCs (5).

DCs do not only play an essential role during host defense but also during chemical-induced immune responses like allergic contact dermatitis (ACD). It is assumed, that about 20% of the general population of Europe and North America are sensitized to at least one contact allergen (6). ACD is a prototypic T cell-mediated delayed-type hypersensitivity immune response that may be elicited after skin contact with organic chemicals or metal ions from cosmetics, jewelery or other commodities. Pathogenesis of ACD is sub-divided in two phases. Upon initial exposure to a contact allergen, the sensitization phase is initiated. Significant clinical symptoms will only emerge after re-exposure to the contact allergen during the so-called elicitation phase (7). The importance of DCs during the sensitization phase is well-understood and also reflected in the adverse outcome pathway on skin sensitization (8). To date, the maturation of DCs in the context of contact allergy has been insufficiently investigated. For instance, there is an ongoing debate, whether or not there are allergy-specific alterations of DCs, which may be used as allergy-specific biomarkers. Proteomics studies can help to elucidate molecular alterations induced by allergens. Proteins directly affect the phenotype of an organism and possess an enormous functional repertoire. They may, for instance, act as transporters, messenger molecules or as enzymatic catalyst. Furthermore, the number of genes is outnumbered by the number of proteins, e.g., due to manifold post-translational modifications. Thus, studying the proteome and metabolism of DCs after exposure to contact allergens is mandatory to get insights in the biological state of the cells (9).

Therefore, the aim of this study was to compare DC maturation induced by the metal allergen NiSO_4_ to the bacterial trigger lipopolysaccharide (LPS). DC maturation by the latter has been investigated extensively (10–13). Nickel has been selected for several reasons. Firstly, nickel is the most common cause of ACD in Europe. Even though numbers of affected individuals are decreasing as the EU nickel directive restricts nickel release from products intended for prolonged skin contact, still 8–18% of the European population are sensitized to nickel (14). Secondly, it was shown that both Ni^2+^ and LPS act via TLR4 although with different molecular binding mechanisms. LPS binds to a specific pocket on myeloid differentiation factor 2 (MD-2) which forms a heterodimer with TLR4 (15), inducing dimerization and internalization of TLR4 (16, 17). In contrast, Ni^2+^ was shown to bind to three histidine residues on the ectodomain of human TLR4, which is distinct from the endotoxin binding site (18). TLR4 dimerization, which is mandatory for receptor activation, was found to occur independently of MD-2 in the presence of Co^2+^ and Ni^2+^ (19).

We were interested in elucidating whether these differences in binding to TLR4 might eventually induce different proteins and pathways. The comparison of an allergen with a bacterial stimulant allows the identification of allergy specific effects and possible biomarkers which may support current *in vitro* testing strategies for skin sensitization. Hence, we here present a global proteomic analysis of LPS and NiSO_4_-treated primary human monocyte-derived DCs (MoDCs) using label-free quantification (LFQ). MoDCs were treated for 24 h with 400 μM NiSO_4_ or 2.5 μg/ml LPS. Upon harvest, cells were prepared for proteomic analysis as well as for flow cytometry analysis of selected cell surface markers and cytokines. Proteomic data were validated using suitable methods like qPCR revealing differences in NiSO_4_- and LPS-induced activation.

## Materials and Methods

### Reagents

Unless stated otherwise, all chemicals were purchased from Sigma-Aldrich. Phosphate buffered saline (PBS, P04-36500), RPMI 1640 (P04-17500), human serum (P-2701), HEPES (P05-01100), sodium pyruvate (P04-43100), penicillin-streptomycin (P06-07100) and non-essential amino acids (NEAA, P08-32100) were purchased from PAN.

### MoDC Generation and Chemical Treatment

PBMCs were isolated by standard density gradient centrifugation with Ficoll Paque Plus (GE Healthcare, 17-1440-03) from human buffy coats. Buffy coats were obtained from German Red Cross (Berlin, Germany) according to the current version of the declaration of Helsinki with an approved ethic vote (Charité, Berlin Germany; EA4/071/13). Untouched CD14^+^ and CD14^+^CD16^+^ monocytes were isolated by negative depletion using the human PAN monocyte isolation kit (Biolegend, 480060), following the manufacturer's instructions. Monocytes were cultured in commercial ready-to-use MoDC differentiation medium containing 400 IU/ml IL-4 and 500 IU/ml GM-CSF (Miltenyi Biotec, 130-094-812) at 37°C with 5% CO_2_. Every two days, half of the culture medium was replaced with fresh medium. On day six, immature MoDCs were harvested, washed with PBS and resuspended in fresh medium at a density of 10^6^ cells/ml. Cells were either treated with medium only as control, 2.5 μg/ml LPS (from Escherichia coli O111:B4, L3024-5MG) or 400 μM NiSO4 (31483, tested endotoxin-free using QCL-1000TM Endpoint Chromogenic LAL Assay (Lonza, 50-647U) according to the manufacturers protocol). For proteomic studies and flow cytometry, cells were harvested after 24 h of continuous chemical incubation; for quantitative RT-PCR, cells were incubated either for 24 h or over a 36 h period with sample collections at various time points.

### Flow Cytometry

To characterize the phenotype of harvested MoDCs, cells were stained for 30 min at 4°C with BV421 anti-CD86 (FUN-1, 562433), FITC anti-CD40 (5C3, 555588), FITC anti–CD83 (HB15e, 556910), PE anti–CD80 (L307.4, 557227), APC anti-CD1a (HI149, 559775) (all BD Biosciences) and PE anti-CD14 (TÜK4, Miltenyi, 130-113-147). Viability was monitored using fixable near-IR dead cell stain (Thermo Fisher Scientific, L34976). For each antibody staining, a control isotype staining was included. Data were acquired using a FACSAria III flow cytometer (BD Biosciences) and analyzed with FlowJo software (V.10.6.1, FlowJo LLC, Ashland, OR, United States). An exemplary gating plot is depicted in Supplementary Figure 1. Graphical visualization was performed using GraphPad Prism 6 (GraphPad Inc., San Diego, CA, United States). On day 6 of MoDC differentiation, the immature state of MoDCs was confirmed by measuring downregulation of the monocytic marker CD14 and upregulation of the DC-marker CD1a (data not shown).

### Phagocytosis of TNBS-Modified Human Serum Albumin

Human serum albumin (HSA, fraction V, Merck, 12668-10GM-M) was dissolved in PBS (10 mg/ml) and incubated at 37°C for 60 min with 5 mM trinitrobenzenesulfonic acid (TNBS, 92822-1ML) (mole ratio HSA:TNBS 1:300) following a modified protocol of Dietz et al. (20). Subsequently, free TNBS was removed with a 30 kDa cutoff spin filter and two consecutive washes with PBS. Purified trinitrophenyl (TNP)-modified HSA was resuspended in PBS, and protein concentration was determined using the Pierce BCA protein assay (Thermo Fisher Scientific, 23225). As a control, pure HSA dissolved in PBS was processed equally. MoDCs were treated for 3 h or 24 h with LPS or 400 μM NiSO_4_. To monitor phagocytosis, cells were harvested, seeded at a density of 10^5^ cells/well in a 96-well plate and incubated with HSA-TNP or pure HSA (200 μg/ml). After 3 h, cells were stained with fixable near-IR dead cell stain followed by an intracellular BV421 anti-TNP (A19-3, BD Biosciences, 562601) stain with inside stain kit (Miltenyi, 130-090-477).

### Mixed Leukocyte Reaction (MLR)

Chemical-treated MoDCs were harvested after 24 h, washed with PBS and 6 × 10^4^ cells/well were seeded in a 96-well U-bottom plate. Following the manufacturer's instructions, allogenic PAN T cells were negatively depleted from PBMCs with PAN T cell isolation kit (Miltenyi, 130-096-535). T cells were labeled with 4 nM CSFE (Thermo Fisher Scientific, C34554) to monitor cell proliferation and added to MoDCs at a concentration of 6 × 10^5^ cells/well (ratio T cells: MoDCs = 10:1). As a negative control, T cells were cultured without MoDCs. As a positive control, a co-culture of T cells and medium-treated MoDCs, was stimulated with 1 μg/ml staphylococcal enterotoxin B superantigen (S4881-1MG). Cells were cultured in RPMI 1640 supplemented with 10% human serum, 2 mM GlutaMAX (Gibco, Thermo Fisher Scientific, 35050061), 10 mM HEPES, 1x NEAA, 1 mM sodium pyruvate, 100 U/ml penicillin, 0.1 mg/ml streptomycin and 100 U/ml β-mercaptoethanol (Gibco, 21985023) at 37°C with 5 % CO_2_. On day 4, proliferating T cells were identified by their reduced CFSE signal.

### Inhibition of p38 and HIF1α

MoDCs were treated with 20 μM SB203580 (Biomol, AG-CR1-0030-M005) to inhibit p38 activity, or with 0.5 nM echinomycin (Biomol, BVT-0267-M001) to inhibit binding of hypoxia-inducible factor (HIF) 1α to hypoxia response elements for 30 min. Subsequently, LPS and NiSO_4_ were added at final concentrations of 2.5 μg/ml and 400 μM, respectively. After 24 h, viability and CD86 expression were analyzed by flow cytometry.

### Immunoblotting of HIF1α

Chemically treated MoDCs were collected after 24 h of treatment. Cells were washed with ice-cold PBS and cell lysis was performed with the lysis buffer described in the LC-MS/MS sample preparation section. Protein concentration was determined using the Pierce BCA protein assay. Subsequently, 20 μg protein were loaded on 10% SDS-PAGEs. Following the separation, proteins were transferred to nitrocellulose membranes (BioRad, 1620115). The immunoblots were blocked in 5% skimmed milk and incubated for 16 h at 4°C to allow binding of the primary anti-HIF1α antibody (H1alpha67, Novus Biologicals, NB100-105). Binding of the secondary antibody (horseradish peroxidase labeled, Dianova, 115-035-206) was performed for 1 h at room temperature. The blot was visualized using Pierce ECL West Pico Substrate (Thermo Fisher Scientific, 34078) using a Fusion FX6 gel documentation (Vilber, Eberhardzell, Germany). A HRP anti-β actin antibody (AC-15, Abcam, ab49900) was used as loading control. Blots were analyzed using the Image Lab 6.0.1 software (BioRad). All values were background-corrected and the HIF1α-signal was normalized to the β-actin signal and medium control.

### Quantification of Secreted Cytokines

MoDC culture supernatants were collected after chemical treatment for 24 h. Cells and debris were removed by centrifugation for 10 min at 300 g. Following the manufacturer's instructions, IL-1β, IL-6, IL-8, IL-10, IL12p70, IL-18 and TNFα were quantified with a customized bead-based LEGENDplex immunoassay (Biolegend) on a FACS Aria III. Data were analyzed using the provided LEGENDplex v8.0 software. Data were log2 transformed and visualized with GraphPad prism. Student's *t*-test was applied to calculate *p*-values relative to medium control.

### Quantitative Real-Time PCR

MoDCs were collected after chemical treatment, washed with ice-cold PBS and resuspended in TRIzol reagent (Invitrogen, Thermo Fisher Scientific, 15596026). RNA was isolated from 10^6^ cells following the TRIzol manufacturer's protocol. Quantification of isolated RNA was performed on a NanoDrop 1000 (VWR, Radnor, PA, United States). One microgram RNA was used for reverse transcription into cDNA using the High-capacity cDNA reverse transcription kit (Applied Biosystems, Thermo Fisher Scientific, 4368813). Quantitative real-time PCR was performed on a TaqMan PCR 7500 fast system (Applied Biosystems) using Fast SYBR green master mix (Applied Biosystems, 4385618) and the following primers: HPRT DNA forward 5′-GTTCTGTGGCCATCTGCTTAG-3′, reverse 5′- GCCCAAAGGGAACTGATAGTC-3′; HIF1α DNA forward 5′-TTTTTGCTGAAGACACAGAAGC-3′, reverse 5′- GCTTGCGGAACTGCTTTCTA-3′; SLC2A1 DNA forward 5′- CCAGCAGCAAGAAGCTGAC-3′, reverse 5′- AGGATGCTCTCCCCATAGC-3′; HMGCR DNA forward 5′-TGTTTACTGGTAACAATAAGATCTGTG-3′, reverse 5′-GTTGACGTAAATTCTGGAACTGG-3′; SOD2 DNA forward 5′-TTGGCCAAGGGAGATGTTAC-3′, reverse 5′-AGTCACGTTTGATGGCTTCC-3′; NQO1 DNA forward 5′-GCACTGATCGTACTGGCTCA-3′, reverse 5′-GAACACTCGCTCAAACCAG-3′; TNFα DNA forward 5′-CTTCTGCCTGCTGCACTTTGGAG-3′, reverse 5′-GGCTACAGGCTTGTCACTCGG-3′; HK2 DNA forward 5′-GTTCCTGGCTCTGGATCTTG-3′, reverse 5′-GGCAATGTGGTCAAACAGC-3′; SREBF2 DNA forward 5′-CACCAAGCACGGAGAGGT-3′, reverse 5′-GGGGAGGAGAGGAAGGAGA-3′; PFKFB3 DNA forward 5′-AAAAGTGTTCAACGTCGGGG-3′, reverse 5'-CGAAAACCGCAATTTGTCCC-3'; SLC2A3 DNA forward 5'-GAGGACGTGGAGAAAACTTGC-3', reverse 5′-GCCAAATTGGAAAGAGCCGA-3′; SLC2A6 DNA forward 5′-ATCCCAGGCATCCTGGTTTG-3′, reverse 5′-GGTCGTTGAGGATCATGGCA-3′. The following genes were analyzed using QuantiTect Primer Assay (Qiagen): HMOX1 (#QT00092645); PRDX1 (#QT01005536); GSTO1 (#QT02394287); CAT1 (#QT00079674). cDNA was 1:10 fold diluted in water and 1 μl was used for qRT-PCR analysis. Gene expression was normalized to the housekeeping gene *HPRT*, and relative gene expression was determined using the ΔΔCT method (21). Significance relative to medium-control was calculated using Student's *t*-test in GraphPad Prism.

### Cholesterol Quantification

To evaluate effects of chemical treatment on cellular cholesterol content, MoDCs were treated with LPS and NiSO_4_ as stated above. As a positive control for the inhibition of the cholesterol biosynthesis, medium-treated MoDCs were treated with 40 μM lovastatin (M2147-25MG). After 24 h, cells were harvested and washed with PBS. Lipids were extracted following the Folch protocol (22). Briefly, cells were resuspended in 500 μL chloroform-methanol (2:1, 1024451000, 1060072500), and lipids were extracted for 20 min by shaking and occasional vortexing at 4°C. Extraction was repeated after addition of 125 μL water. The suspension was centrifuged for 10 min at 1,000 g and 300 μL of the organic phase were transferred to a new Eppendorf tube and vacuum-dried. Proteins were obtained from the interphase and their concentration was determined using the Pierce BCA protein assay. Cholesterol levels of the samples were quantified using the Amplex Red Cholesterol Assay Kit (Thermo Fisher Scientific, 10236962). Desiccated lipids were dissolved in assay buffer and analyzed following the manufacturer's protocol. Fluorescence was measured at 590 nm emission on a Synergy Neo2 plate reader (BioTek, Winooski, VT, United States) with excitation at 540 nm. Total cholesterol levels were standardized to the respective protein concentration and normalized to the untreated control. *P*-values were calculated using Student's *t*-test relative to the medium control.

### LC-MS/MS

#### Cell Lysis

After chemical treatment, MoDCs were collected, washed twice with ice-cold 0.9% NaCl and lysed in 100 μL lysis buffer per 10^6^ cells. The lysis buffer was composed of 150 mM NaCl (S7653-250G), 10 mM TRIS pH 7.2 (T1503-250G), 5 mM EDTA (E5134-250G), 0.1% SDS (436143-25G), 1% Triton X-100 (T8787-100ML), 1% sodium deoxycholate (30970-100G), 200 μM phenylmethylsulfonyl fluoride (P7626-1G), 1 mM sodium orthovanadate (S6508-10G) and cOmplete protease inhibitor cocktail (Roche, 1.167.498.001). Cells were vortexed, incubated on ice for 15 min and centrifuged. The protein concentration of the supernatant was determined using Pierce BCA protein assay.

#### Sample Preparation

An untargeted proteomics approach was applied using label-free quantification as described before (23). Briefly, 30 μg protein per sample was reduced with 0.1 μmol tris(2-carboxyethyl) phosphine (C4706-10G), followed by alkylation with 0.2 μmol iodoacetamide (Merck, 8.04744.0025). Protein solutions were acidified and acetonitrile (ACN, Carl Roth, AE70.2) was added to reach more than 50 % (v/v) organic content facilitating protein binding to SpeedBeads™ magnetic carboxylate modified particles (SP3 beads, GE Healthcare, 65152105050250). Proteins were loaded on 20 μg beads, followed by a first washing step with 70% (v/v) ethanol (Merck, 22.462.500) in water and a second step with 100 % (v/v) ACN. Proteins were digested with trypsin (enzyme:protein ratio 1:50, Promega, V5117) in 100 mM tetraethylammonium bromide (T7408-100ML). Digestion was stopped by addition of 100 % (v/v) ACN to reach ≥ 95% (v/v) organic content, thus again allowing the peptides to bind to the beads. Peptides were again cleaned-up using 100% (v/v) ACN. Elution of peptides was carried out in two steps with 87% (v/v) ACN in ammonium formate (pH 10) (Agilent Technologies, AGG1946-85021), followed by 2% (v/v) dimethyl sulfoxide (D2650-5x-10ml), resulting in two fractions per sample. Samples were evaporated to dryness and reconstituted in 0.1% (v/v) formic acid (FA, Fluka, Thermo Fisher Scientific, 56302-50ML-F) before measurement.

#### LC-MS/MS

Samples were analyzed on a UPLC system (Ultimate 3000, Dionex, Thermo Fisher Scientific) coupled to a Q Exactive HF (Thermo Fisher Scientific) as described previously (23). Peptides were loaded on an Acclaim PepMap 100 C18 trap column (3 μm, nanoViper, 75 μm × 5 cm, Thermo Fisher Scientific, PN164535) at a flow rate of 5 μl/min using an eluent composed of 2% (v/v) ACN and 0.05% (v/v) trifluoroacetic acid (Biosolve, 202341A8) in water. Peptides were separated by a 150 min non-linear gradient from 0 to 80% ACN in 0.1% FA on a reversed-phase column (Acclaim PepMap 100 C18, 3 μm, nanoViper, 75 μm × 25 cm, Thermo Fisher Scientific, PN164569). A chip-based ESI source (Nanomate, Advion, Ithaca, NY, United States) was used for ionization at 1.7 kV and coupled to the Q Exactive HF. The MS1 scans were acquired at a resolution of 120K in a range of 350–1,550 *m/z*. AGC target was set to 3 × 10^6^ with a maximal injection time of 100 ms. The top 10 most abundant peptides were isolated for MS2 acquisition with an isolation window of 1.4 *m/z*. Peptides were fragmented at normalized collision energy of 28, and the fragment ion spectra were acquired at a resolution of 15K using AGC target of 2 × 10^5^ and maximal IT of 100 ms. All spectra were acquired using XCalibur (Version 3.0).

#### Analysis

MS raw data were processed with MaxQuant Version 1.6.3.10 (24). If not stated otherwise, default parameters were used. Peptides were identified using a database search against the *Homo sapiens* UniProtKB reference proteome (24-09-2019, 74349 reviewed and unreviewed entries). Carbamidomethylation of cysteine was chosen as fixed, whereas oxidation of methionine and acetylation of protein N-terminus were set as variable modifications. A minimum of two peptides with at least one unique peptide was required for protein identification applying FDR ≤ 0.01. Match between runs was activated. Proteins were quantified based on two unique peptides. Protein contaminants and reverse hits were excluded before further use. LFQ protein intensities were processed, and results were visualized in R-3.5.0. Accordingly, the data were log2-transformed, filtered for proteins that were quantified in a minimum of four replicates under at least one condition, followed by variance-stabilization. Imputation was performed using the DEP package (25) (fun = “MinProb,” *q* = 0.01) for proteins not quantified in any of the replicates under the particular condition. Fold changes (FCs) and *p*-values were calculated using Student's *t*-test relative to the medium control. Proteins with a *p*-value ≤ 0.05 were considered as significantly changed.

#### Pathway Enrichment

Significantly enriched pathways were identified using ingenuity pathway analysis (IPA, Qiagen), considering significantly enriched proteins.

## Results

### Phenotypical and Functional MoDC Characterization

The effects of NiSO_4_ and LPS on the phenotype and function of primary human monocyte-derived DCs (MoDCs) were investigated first. MoDCs were generated from buffy coats of five individual donors, and treated with LPS (2.5 μg/mL) or NiSO_4_ (400 μM) for 24 h. Cell viability and upregulation of the maturation markers CD86, CD83, CD40 and CD80 on their surface was measured (Supplementary Figure 1). Cell viability was assured to be above 75% for all samples (Supplementary Figure 1A), in agreement with the acceptance criteria of the human Cell Line Activation Test (26). MoDCs were used in further experiments, if maturation markers were upregulated after LPS- and NiSO_4_-treatment (Supplementary Figures 1B–E). Overall, changes induced by LPS were more pronounced.

Furthermore, the gene expression of the allergy relevant cytokine TNFα was determined by qRT-PCR. The *TNFA* gene is directly induced after TLR4 signaling and activates nuclear factor κB (NFκB). To capture the dynamics of *TNFA* expression, we measured its gene expression over 36 h at various time points. The time course of gene induction was comparable for LPS and NiSO_4_ peaking 2 h after treatment. However, LPS acted as a much more potent inducer (Supplementary Figure 2).

Moreover, selected inflammatory cytokines (IL-1β, IL-6, IL-8, IL-10, IL12p70, IL-18, and TNFα) were quantified in the cell culture supernatants using a bead-based immunoassay (Supplementary Figure 3). The production of IL-8 for example is used as a biomarker for DC activation (26). All cytokines were significantly enriched in the cell culture supernatant of both treatments compared to the unstimulated control. Only for IL-10, NiSO_4_-induced release was not significantly altered compared to medium control. Overall, the secreted amounts of all analyzed cytokines were higher in LPS-treated compared to NiSO_4_-treated cells.

#### The Phagocytotic Activity of LPS- and NiSO_4_-Treated MoDCs Is Markedly Reduced

It is widely accepted that mature DCs are less phagocytotic compared to their immature progenitors. The degree of reduction of phagocytosis depends on the stimuli as well as on the DC maturation state (27–29). Thus, we aimed to functionally compare dendritic cells that were maturated with either LPS or NiSO_4_ by monitoring uptake of trinitrophenyl-modified HSA. MoDCs maturated for 3 h with either substance showed no significant reduced phagocytotic activity compared to untreated cells (Supplementary Figure 1G). However, prolonged chemical treatment of 24 h reduced phagocytosis of MoDCs (Supplementary Figure 1F). The degree of reduction in phagocytotic activity was comparable for LPS and NiSO_4_.

#### LPS- and NiSO_4_-Treated MoDCs Induce Proliferation of Allogenic T Cells

To test whether NiSO_4_ affects the ability of MoDCs to activate T cells, we performed a mixed leukocyte reaction. Control, LPS- or NiSO_4_-treated MoDCs were incubated for 4 days with allogenic T cells, and proliferation of the latter was determined by CFSE dilution. LPS- and NiSO_4_-treated MoDCs induced stronger proliferation of T cells compared to control MoDCs with LPS being the more potent inducer (Supplementary Figure 4A). These results are in concordance with the upregulation of co-stimulatory molecules on the DC surface. As control, T cells were incubated in the absence of MoDCs, which resulted in hardly any proliferation compared to T cells cultured in the MLR (negative control, data not shown) while the superantigen staphylococcal enterotoxin B induced a strong T cell proliferation (positive control, data not shown).

Overall, the upregulation of activation markers, expression of inflammatory cytokines, reduced antigen-uptake and the ability to induce T cell proliferation prove the activation of MoDCs after treatment with NiSO_4_ and LPS. Thus, even if less pronounced compared to LPS, immunological signaling induced by NiSO_4_ was demonstrated.

### Proteomic Analysis

**Figure 1:**
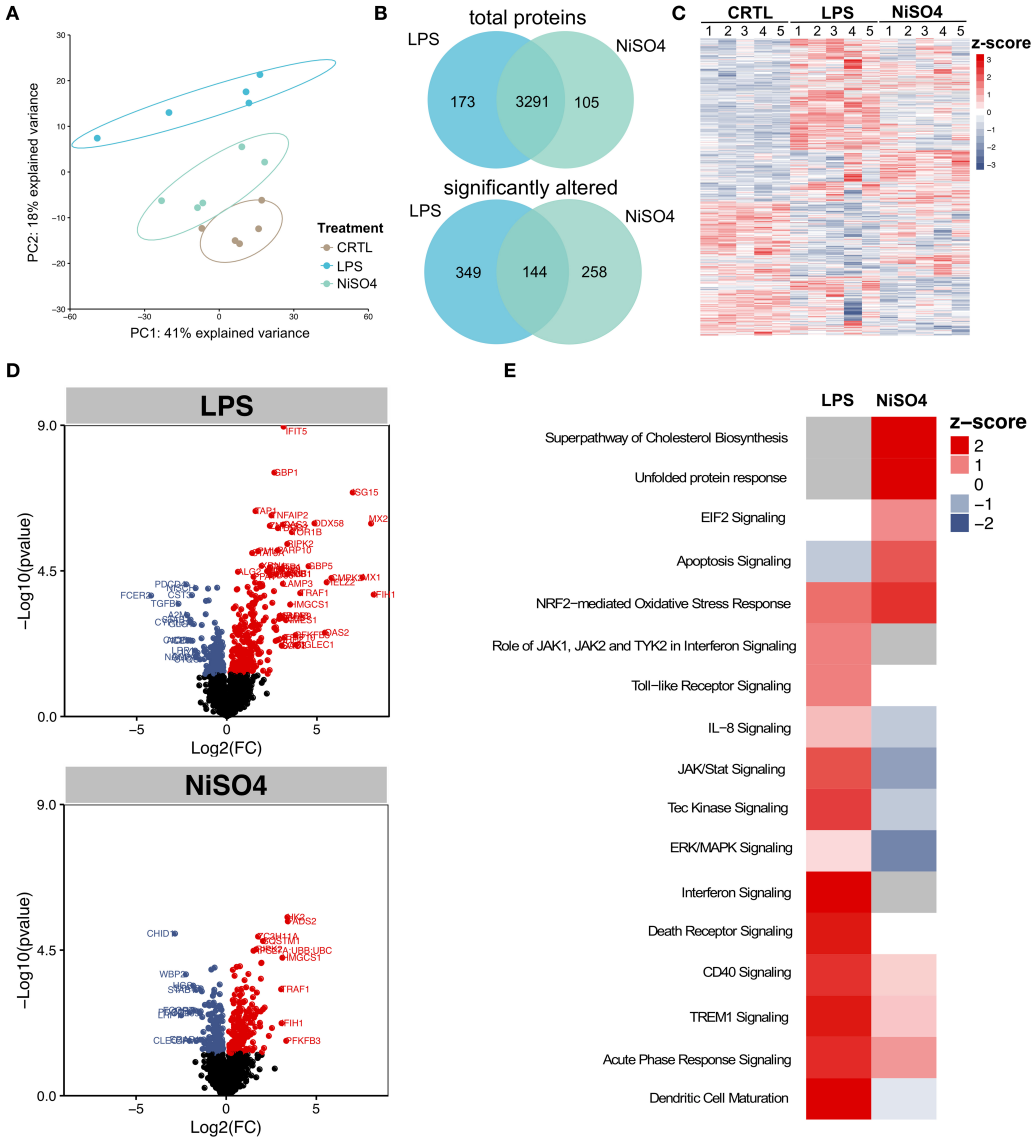
Quantitative proteomic analysis of differentially regulated proteins in LPS- and NiSO_4_-treated MoDCs. **(A)** Principal component analysis of the five biological replicates showing a distinct clustering of each group, **(B)** Venn-diagrams of total as well as significantly altered proteins in the LPS-group (blue) and NiSO_4_-group (green), **(C)** heat map of significantly regulated proteins. Plotted are z-scores for all identified proteins, **(D)** volcano plots showing significantly regulated proteins for LPS- and NiSO_4_-treated MoDCs compared to untreated MoDCs (*p* ≤ 0.05). Mean values of the log2-fold change are displayed and plotted against –log10(*p*-value), red = upregulation, blue = downregulation, **(E)** significantly changed pathways in LPS and NiSO_4_-treated MoDCs. Canonical IPA pathways with a positive z-score (red) are predicted to be activated; blue represents a negative z-score and inactivation of the pathway. Gray indicates unmatched pathways. IPA analysis was based on proteins that were significantly changed compared to control (*p* ≤ 0.05). Within the IPA software, z-scores were calculated and only experimental data of human origin was allowed. The tissue specificity was set to immune cells. Significance was calculated using Student's *t*-test.

To analyze the underlying cellular effects and to identify similarities and changes of LPS- and NiSO_4_-activated DCs, we applied a global proteomics approach. A principal component analysis was employed to assess the similarity of the treatment groups. Biological replicates show little variance, indicative for the high quality of our proteomic dataset. NiSO_4_-samples cluster between LPS- and control-samples (Figure 1A), pointing toward an intermediate cellular state of NiSO_4_-treated cells. In total, more than 3,300 proteins were identified using a label-free quantification approach (Supplementary Table 1). Of these, the majority was identified in both treatment groups (Figure 1B). Analysis of significantly altered proteins relative to the control exhibited 493 significantly altered proteins by LPS, while 402 proteins were changed by NiSO_4_-treatment and 144 proteins were altered by both treatments with *p* ≤ 0.05 (Figure 1B). Furthermore, the z-score of each fold change was calculated and plotted in a heat map (Figure 1C), which allowed comparison across replicates and treatments. Biological replicates show minimal variance, which allowed the identification of clusters of significantly altered proteins compared to the control. Interestingly, we observed different clusters of upregulated proteins for NiSO_4_-treated cells compared to LPS-treatments, indicating a distinct mode of action. Next, we visualized the data using volcano plots to identify statistically significantly regulated proteins based on their *p*-value compared to the magnitude of change. As shown in Figure 1D, LPS induced a more pronounced spread of the data. Overall, upregulation was favored over downregulation for both chemicals.

To differentiate the effects of LPS- or NiSO_4_-treatment on cellular pathways, significantly altered proteins (*p* ≤ 0.05) were used to identify enriched pathways using QIAGEN's Ingenuity Pathway Analysis (IPA). Notably, LPS-treatment induced more significantly enriched pathways than NiSO_4_ (Figure 1E). Shared pathways between the two groups included Nrf2-mediated oxidative stress response, CD40 signaling, triggering receptor expressed on myeloid cells (TREM) 1 signaling and acute phase response signaling. Pathways that were uniquely induced after NiSO_4_-treatment comprised the superpathway of cholesterol biosynthesis, the unfolded protein response and eukaryotic initiation factor (EIF) 2 signaling.

### NiSO_4_-Treatment Activates Nrf2 Target Genes in MoDCs

**Figure 2:**
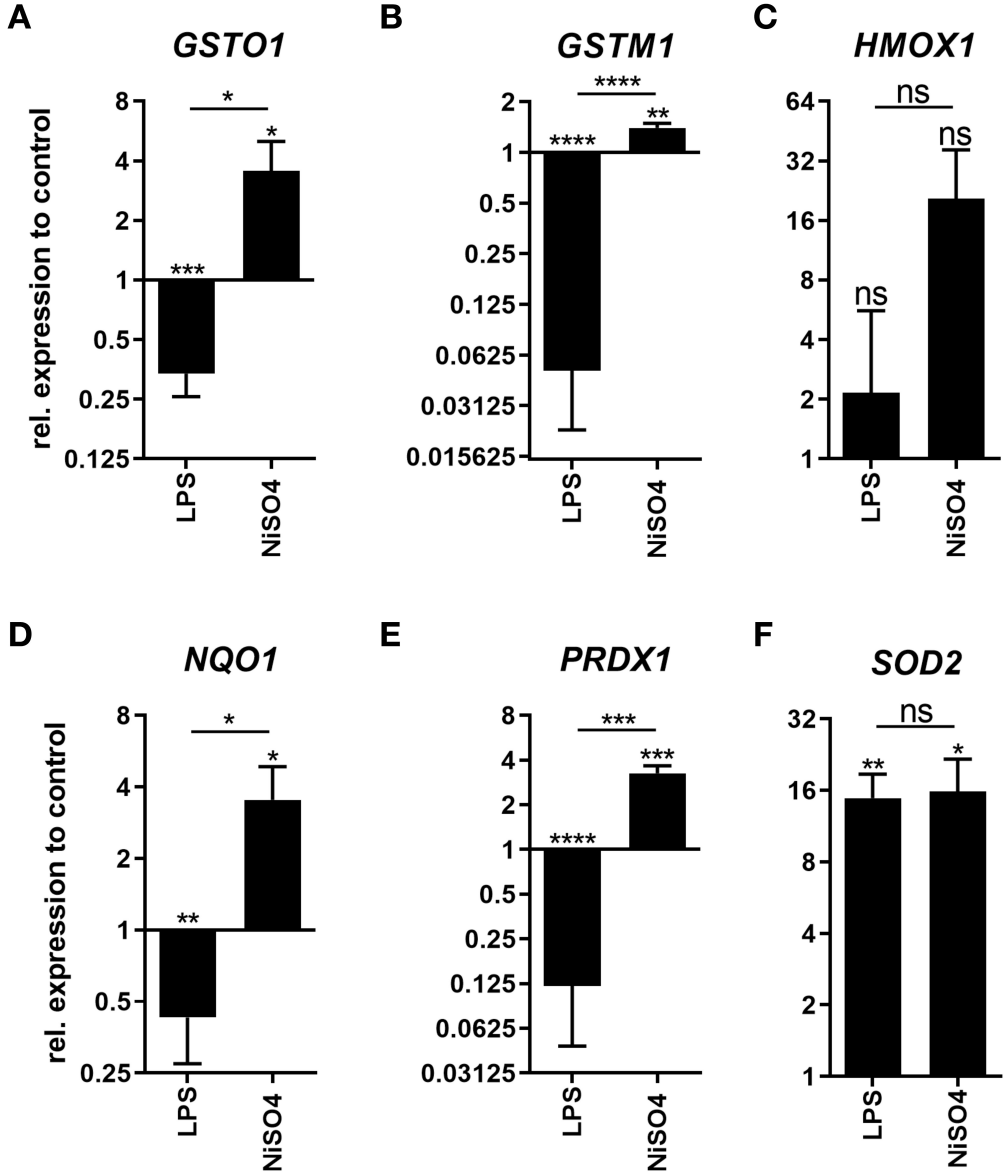
Expression of Nrf2-target genes in LPS- and NiSO_4_-treated MoDCs. Depicted is the gene expression of glutathione S-transferase omega (GSTO1, **A**), glutathione S-transferase mu (GSTM1, **B**), heme oxygenase (HMOX1, **C**), NAD(P)H dehydrogenase (NQO1, **D**), peroxiredoxin (PRDX1, **E**) and superoxide dismutase (SOD2, **F**) as measured by the qRT-PCR after treatment of MoDCs with medium only, 2.5 μg/ml LPS or 400 μM NiSO_4_ for 24 h. Gene expression was normalized to the gene expression of the housekeeping gene HPRT and medium control. Thereof, mean and SD are shown (*n* = 3 donors). Applying the Student's *t*-test, significance was calculated to medium control (*p-value ≤ 0.05; **p-value ≤ 0.01; ***p-value ≤ 0.001; ****p-value < 0.0001).

The nuclear factor erythroid 2-related factor 2 (Nrf2)-mediated oxidative stress response was predicted to be highly activated in the ingenuity pathway analysis for NiSO_4_-treated MoDCs. Furthermore, this pathway was among the few pathways that were induced much stronger by NiSO_4_ compared to LPS (Figure 1E). Hence, we ought to investigate the differences between the two stimuli concerning the Nrf2 pathway in more detail. Gene expression of Nrf2-target genes was analyzed by quantitative RT-PCR (Figure 2). Indeed, 4 out of 6 genes, namely *GSTO1, GSTM1, NQO1*, and *PRDX1*, were induced only due to NiSO_4_- but not after LPS-treatment. *HMOX1* gene expression was more pronounced after NiSO_4_-treatment; however, the effect was not statistically significant. *SOD2* gene levels were exacerbated after both treatments, rendering *SOD2* the only Nrf2 target gene that was found to be significantly upregulated after LPS-treatment. On the protein level, heme oxygenase (HMOX) 1 was confirmed in the proteomic data set as significantly upregulated after NiSO_4_-treatment. Superoxide dismutase (SOD) 2 showed strong upregulation on protein level after both treatments. However, only LPS induced changes were significant. From this data, it can be concluded that NiSO_4_ causes a distinct oxidative stress level in MoDCs which may strongly contribute to the activation of the cells. Thus, induction of oxidative stress may be an additional mode of action besides TLR4 activation in NiSO_4_-treated MoDCs.

### NiSO_4_ Leads to Cholesterol Depletion but Induces Cholesterol Biosynthesis Pathway

**Figure 3:**
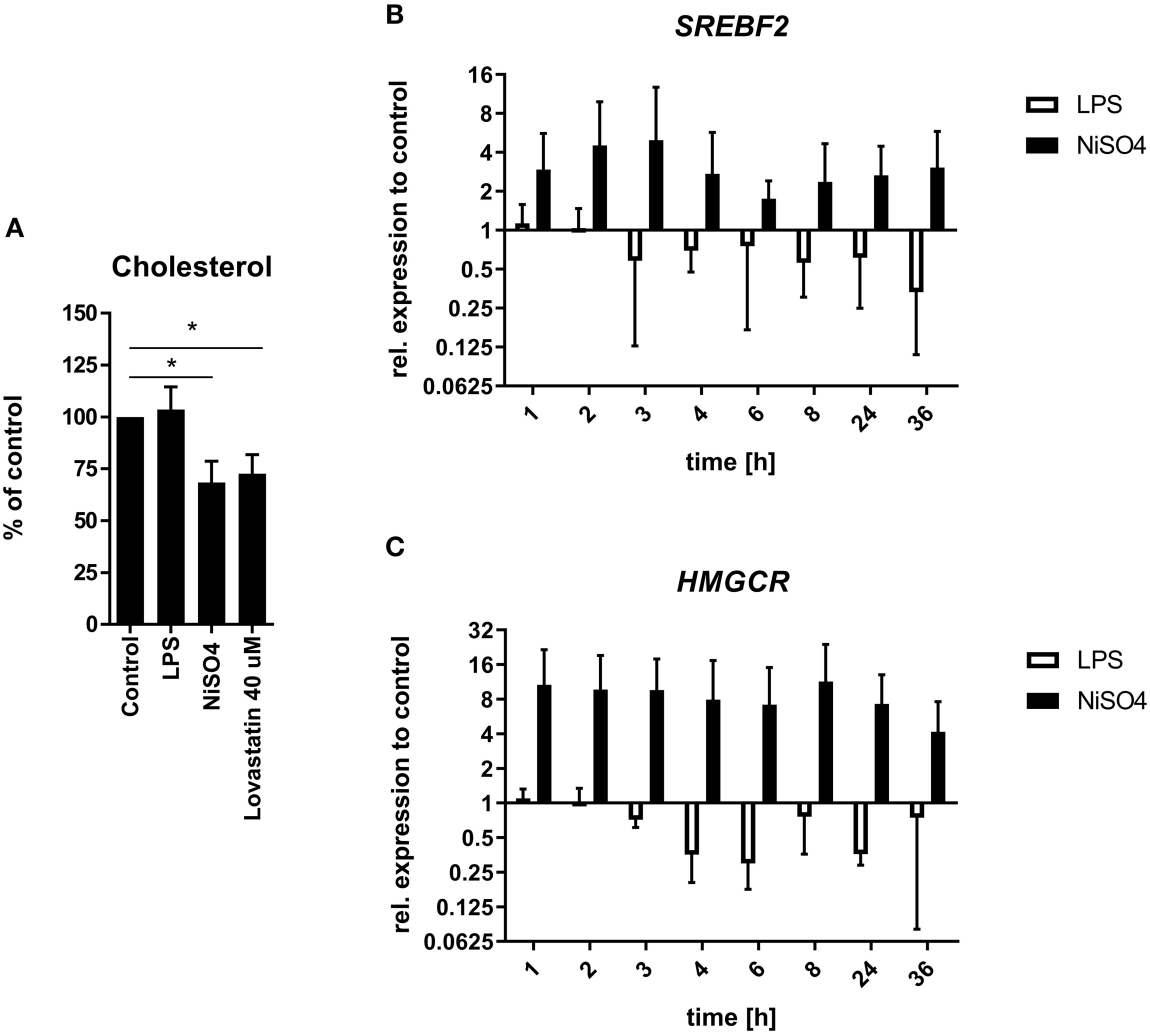
Cellular cholesterol quantification and relative gene expression of relevant genes in LPS- and NiSO_4_-treated MoDCs. **(A)** Cellular cholesterol levels were determined fluorometrically using the Amplex Red Cholesterol Assay kit after lipid extraction from MoDCs treated for 24 h. The fluorescence intensity was normalized to the protein concentration of the respective sample. Cholesterol/protein-ratios were then further normalized to the untreated control. Significance to medium control was calculated using Student's *t*-test and is indicated with *p ≤ 0.05. The relative gene expression of sterol regulatory element-binding protein 2 (SREBF2, **B**) as well as 3-hydroxy-3-methylglutaryl-coenzyme A reductase (HMGCR, **C**) were analyzed at selected time points after treatment of MoDCs. Gene expression was measured by qRT-PCR and normalized to the gene expression of HPRT (housekeeping gene) and medium control. Plotted are mean and SD of the normalized data (*n* = 3 donors). For each assay MoDCs were treated with medium only, 2.5 μg/ml LPS, 400 μM NiSO_4_ or 40 μM lovastatin.

Pathway enrichment analysis by IPA revealed the superpathway of cholesterol biosynthesis as one of the strongly activated pathways after treatment of MoDCs with NiSO_4_ (Figure 1E). Proteins matched to this pathway were significantly upregulated and comprised proteins like lanosterol 14α-demethylase (CYP51A1), hydroxymethylglutaryl-CoA synthase **(**HMGCS1) and 7-dehydrocholesterol reductase. We therefore quantified cellular cholesterol levels using the Amplex Red Cholesterol assay and confirmed a significant depletion of cholesterol in NiSO_4_-treated MoDCs (Figure 3A). The strength of this effect was comparable to that of cells treated with lovastatin, an inhibitor of 3-hydroxy-3-methyl-glutaryl-coenzyme A reductase **(**HMGCR), which is the rate-limiting enzyme of the cholesterol biosynthesis. In contrast, LPS did not induce changes in cellular cholesterol levels. Based on these findings, we analyzed the gene expression of HMGCR and sterol regulatory element-binding protein (SREBB) 2, the regulating transcription factor of this pathway. Both genes were found to be constantly upregulated in NiSO_4_-treated MoDCs but were not induced (or even slightly downregulated) after treatment with LPS (Figures 3B,C).

### LPS and NiSO_4_ Induce Metabolic Shifts That Lead to Hypoxic Conditions in Case of NiSO_4_

**Figure 4:**
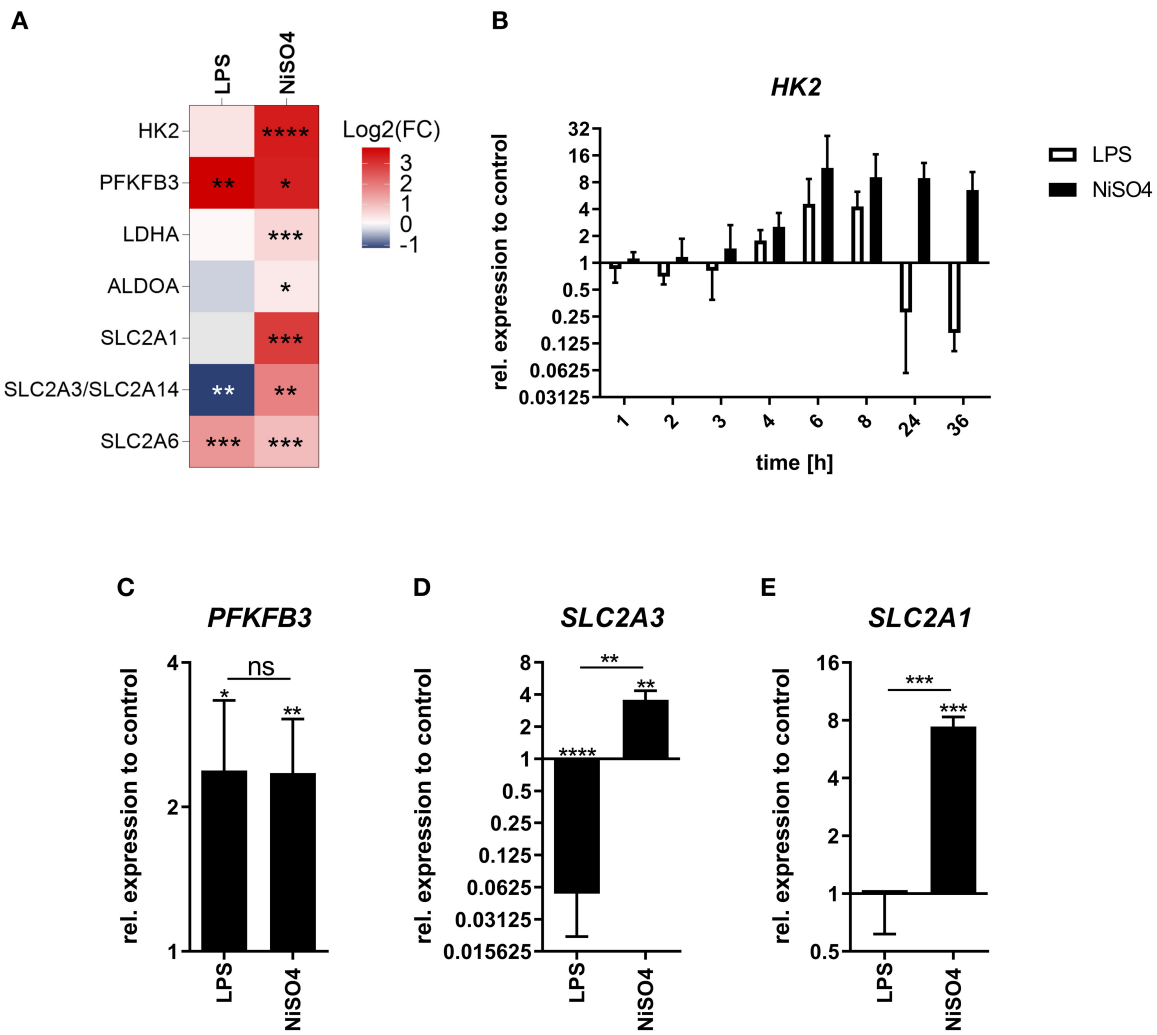
Fold change of glycolytic proteins and their respective gene expression in LPS- and NiSO_4_-treated MoDCs. **(A)** Heat map of differentially upregulated proteins in the proteomics data set linked to glycolysis and glucose transport. Red indicates upregulation of the respective protein and blue refers to downregulation (*n* = 5 donors). **(B–E)** Relative gene expression of hexokinase 2 (HK2, B), 6-phosphofructo-2-kinase/fructose-2,6-bisphosphatase 3 (PFKFB3, C), solute carrier family 2 member 3 (SLC2A3, D) and solute carrier family 2 member 1 (SLC2A1, E). Gene expression was measured by qRT-PCR at indicated time points **(B)** or after 24 h of treatment for **(C–E)**. Gene expression was normalized to the gene expression of HPRT (housekeeping gene) and medium control. Mean and SD of the normalized data are shown (*n* = 3 donors). Significance to medium control was calculated using Student's *t*-test (ns: not significant; *p-value ≤ 0.05; **p-value ≤ 0.01; ***p-value ≤ 0.001; ****p-value < 0.0001). MoDCs were treated with medium only, 2.5 μg/ml LPS or 400 μM NiSO_4_.

Metabolic reprogramming is essential during DC maturation since the metabolic switch from oxidative phosphorylation to glycolysis ensures adequate energy supply to support cellular reconstructions. Thus, we aimed to investigate the effects of NiSO_4_ on the metabolism of DCs. In the proteomic data set, proteins associated with anaerobic glycolysis were significantly upregulated after treatment with NiSO_4_, including lactate dehydrogenase (LDHA) along with the glycolytic enzymes aldolase A (ALDOA), hexokinase (HK) 2 and 6-phosphofructo-2-kinase/fructose-2,6-biphosphatase (PFKFB) 3. These findings were supported by elevated expressions of proteins from the glucose transporter (GLUT) family which facilitate the transport of sugars. Namely, solute carrier (SLC) 2A1, SLC2A3/2A14 and SLC2A6 expression were significantly increased after NiSO_4_-treatment (Figure 4A).

In contrast, cells treated with LPS induced only upregulation of PFKFB3 and SLC2A6. SLC2A1 was not detected, and SLC2A3 was significantly downregulated in this group (Figure 4A). Thus, we aimed to investigate these differences in more detail using RT-PCR. Gene expression analysis suggests different kinetics for *HK2* gene expression. Both treatments resulted in an increasing *HK2* gene expression over the first 6 h of treatment. After 6 h a plateau was reached, which NiSO_4_-treated MoDCs maintained over the investigated time. Yet, LPS-treated MoDCs appear to counteract this gene induction leading to subsequent downregulated expression of *HK2* (Figure 4B). Upregulation of PFKFB3 induced by LPS and NiSO_4_ was verified by measuring gene expression after 24 h (Figure 4C). Protein expression of the GLUT family proteins was confirmed by measuring the gene expression of the respective genes 24 h after treatment (Figures 4D,E, Supplementary Figure 5B). Stimulation of MoDCs with LPS was shown to cause metabolic reprogramming that was mainly dependent on p38 mitogen-activated protein kinase signaling leading to HIF1α accumulation and elevated HK2 gene expression and activity (30). In our study, expression of the *HIF1A* gene was downregulated for both groups (Supplementary Figure 5A) but quantitative immunoblot analysis revealed increased cellular HIF1α protein levels after 24 h of treatment (Supplementary Figure 5C). Since we found HK2 upregulated on protein and gene level, we were interested in studying whether inhibition of p38 using the inhibitor SB203580 had similar effects in LPS- and NiSO_4_-stimulated cells. Indeed, CD86 expression was decreased after inhibition of p38. However, the reduction was only significant in LPS-treated MoDCs (Supplementary Figure 6A). Additionally, we analyzed the effect of echinomycin-induced HIF1α inhibition on the MoDCs. Inhibition of HIF1α induced a significant reduction of the CD86 fluorescence signal in NiSO_4_- treated cells (Supplementary Figure 6B). The pronounced dependency of NiSO_4_-treated cells on HIF1α along with increased protein levels and gene expression of glycolytic enzymes and glucose transporters can be indicative for a prolonged stabilization of HIF1α and hypoxia-like conditions (31).

**Figure 5:**
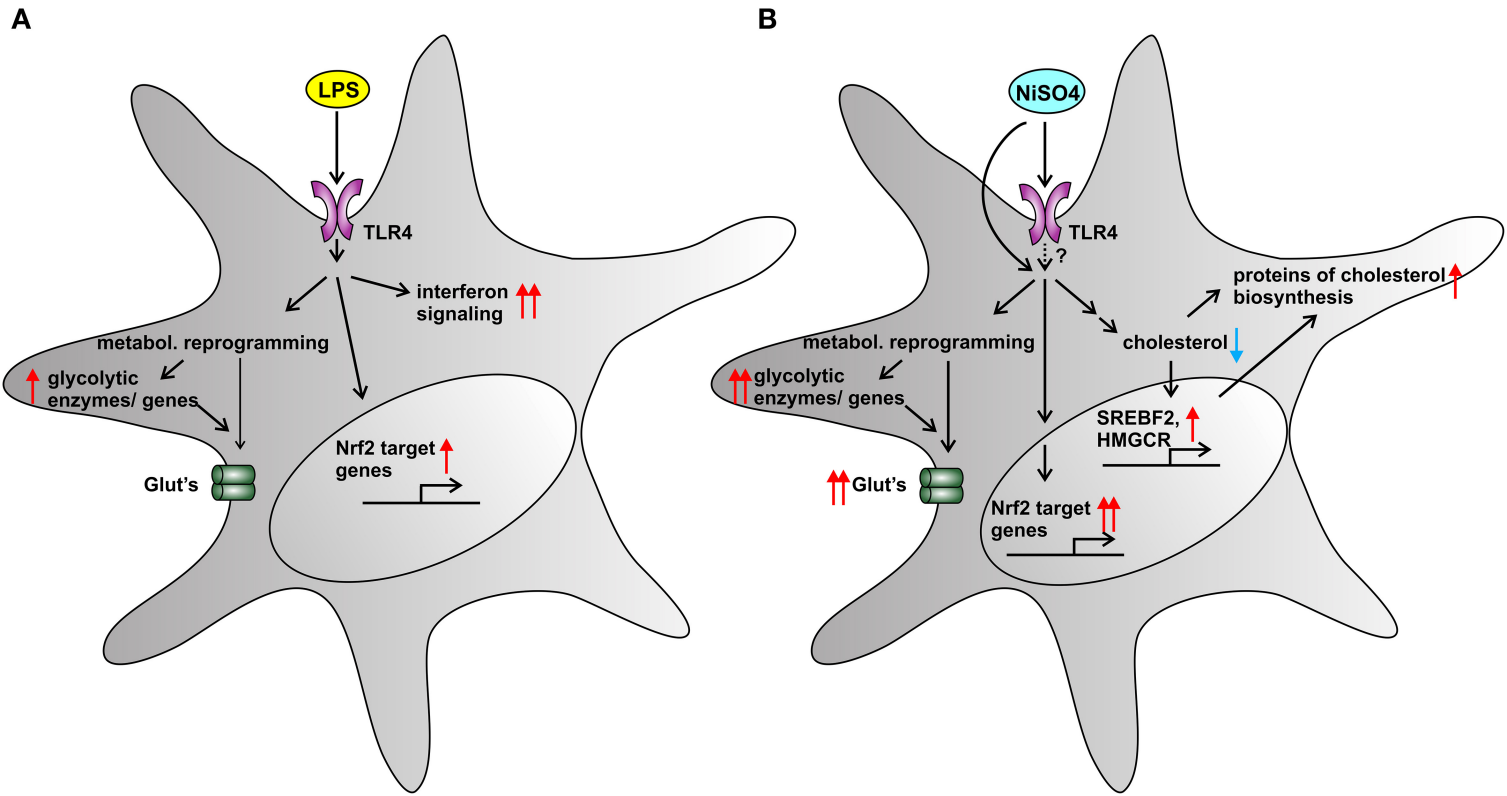
Summarizing Figure. Cellular effects of two TLR4-activators on MoDCs: LPS **(A)** and NiSO_4_ **(B)**. **(A)** LPS-treatment induced a metabolic shift toward glycolysis and a weak Nrf2-dependent stress response. IPA pathway analysis revealed a strong upregulation of interferon signaling. **(B)** NiSO_4_-treated cells showed a distinct upregulation of glycolytic enzymes both on mRNA and protein level, suggesting metabolic reprogramming and hypoxia-like conditions. The metal allergen NiSO_4_ also triggered a pronounced Nrf2-dependent stress response that culminated in upregulation of Nrf2-target genes. Most strikingly, NiSO_4_ induced a cellular cholesterol depletion in MoDCs, which was reflected in the upregulation of genes and proteins involved into cholesterol biosynthesis. We speculate that haptenation to other cellular proteins than TLR4 also contributed to the observed changes. A blue arrow indicates downregulation and red arrows indicate upregulation. A pronounced change is indicated by two arrows.

Figure 5 summarizes the main findings of this study in a comprehensive and visual way.

## Discussion

The present study aimed at elucidating the cellular mode of action of NiSO_4_-stimulation in primary human MoDCs in comparison to LPS activation. Nickel is a well-known allergen, whereas LPS belongs to pathogen-associated molecular patterns of Gram-negative bacteria. Both agents are known to activate DCs by ligation to TLR4 (15, 18). As the molecular binding mechanism is not identical (19), we studied if this may lead to the differential regulation of allergy-specific proteins in NiSO_4_-treated MoDCs. To investigate proteome alterations and underlying mechanisms, we applied different approaches including proteomics, quantitative RT-PCR and flow cytometry. MoDCs generated from five individual donors were treated with 2.5 μg/ml LPS or 400 μM NiSO_4_ to induce maturation of DCs. We confirmed maturation of MoDCs by both stimuli along with reduced phagocytosis, secretion of immunostimulatory cytokines as well as induction of T cell proliferation. Overall, NiSO_4_-treatment appears to elicit a phenotype comparable to LPS.

At the protein level, LPS-treated cells in our study elicited proteins and signaling as reported previously. Arya et al. (13) conducted a study to characterize proteomic changes induced in MoDCs after LPS treatment. The pathways of interferon signaling and IL-9 signaling and the proteins nuclear factor NF-kappa-B p105 subunit (NFKB1), nuclear factor NF-kappa-B p100 subunit (NFKB2), IL-1β, TNF receptor-associated factor (TRAF) 1, E3 ubiquitin-protein ligase TRIM4, CD54, fascin and signal transducer and activator of transcription 1 were also significantly altered by LPS in our study. NiSO_4_-treatment likewise lead to significantly altered levels of NFKB2, fascin, CD54 and TRAF1. Hussaarts et al. (3) analyzed proteomic changes in MoDCs that were incubated with LPS for 32 h. The authors identified TRAF1, myristoylated alanine-rich C-kinase substrate, human leukocyte antigen B as well as fascin as TOP4 upregulated proteins. We could confirm these findings for LPS-treated MoDCs. However, after NiSO_4_-treatment, only TRAF1 and fascin were significantly altered. The respective TOP4 downregulated proteins in the study by Hussaarts et al. (3) were cathepsins, ganglioside GM2 activator and macrophage mannose receptor 1, which again were also significantly downregulated after LPS-treatment in our proteomic data. Macrophage mannose receptor 1 was the sole protein that was significantly downregulated after NiSO_4_-treatment, too. Despite these eight proteins, also other proteins were identified to be significantly regulated after LPS-treatment in both studies, such as SOD2, WARS and CD54 indicating that the maturation of MoDCs due to LPS is generally robust. In the context of allergy, Strasser et al. (32) published a proteomic study in MoDCs reacting to the prominent birch pollen allergen Bet v 1. The pure recombinant allergen induced only minor changes in MoDC. However, changes caused by birch pollen extract were comparable to LPS induced changes. The authors therefore suspect that Bet v 1 only displays its allergenic potential in combination with additional danger signals such as LPS. The data presented for the birch pollen extract and LPS show high similarities with the data set presented here. The allergenic pollen extract and NiSO_4_ induce similar IPA pathways including Nrf2 mediated oxidative stress response, TREM1 signaling and ERK/MAPK signaling. Furthermore, we were able to identify HMOX1, SOD2, sequestosome-1, TRAF1 and nicotinamide phosphoribosyltransferase as significantly changed due to NiSO_4_. Proteomic changes induced by the investigated NiSO_4_ are hence more comparable with the birch pollen extract than with the recombinant protein itself. It can be suspected that NiSO_4_ does possess the ability to induce danger signaling that Bet v 1 seems to lack. These findings also point to TREM1 as one important signaling pathway in the induction of an allergy.

In our study, both stimuli, LPS and NiSO_4_, induced protein and gene expression changes that are characteristic for TLR4 signaling, reflected by upregulation of NFκB2 on the protein level. NFκB signaling was identified as a crucial pathway in DC maturation in response to NiSO_4_ as it is tightly connected to the expression of maturation markers like CD40, HLA-DR, CD86 and the secretion of pro-inflammatory cytokines like IL-8 and IL-6 (33). Furthermore, NFκB signaling is indicative for the activation of TLR4 by NiSO_4_. The central role of NFκB in mediating the response to NiSO_4_ in MoDCs was also reflected in the strong upregulation of proteins such as SAM and SH3 domain-containing protein (SASH) 1 and TRAF1 which are known to positively regulate NFκB activity (34, 35) and which were also significantly upregulated in our proteomic data set for NiSO_4_-exposed MoDCs. Downstream of NFκB, we identified increased gene expression for TNFα and could also show that NiSO_4_ triggered elevated secretion of this cytokine along with IL-8. Again, LPS induced more pronounced alterations, suggesting a more substantial capacity to activate TLR4 compared to NiSO_4_.

One of the most distinct differences between LPS- and NiSO_4_-treatment was the induction of the Nrf2-pathway after treatment with NiSO_4_. Nrf2 is an important transcription factor for cytoprotective genes. Under normal conditions, Nrf2 is located in the cytoplasm where it is associated with a protein complex including the kelch-like ECH-associated protein **(**KEAP) 1. KEAP1 continually promotes ubiquitination and proteasomal degradation of Nrf2 (36). Electrophilic and oxidative stress induces a conformational change in KEAP1 which suppresses Nrf2 ubiquitination and enables translocation of Nrf2 to the nucleus. In the nucleus, Nrf2 binds to antioxidant-responsive elements and induces transcription of target genes (37). Nrf2-dependent stress response was identified as a major player in the immunological reaction to allergens (38). Specifically, the high relevance of Nrf2 during skin sensitization was reflected in the upregulation of target genes like HMOX1 and NAD(P)H dehydrogenase [quinone] (NQO) 1 at the protein level in CD34-derived dendritic cells and THP-1 cells (39). A prior proteomic study of our lab used Nrf2 knockout mice for the identification of potential biomarkers for skin sensitization among them HMOX1 and PRDX1 (40). Previously, activation of Nrf2 due to treatment with NiCl_2_ was shown in monocytic THP-1 cells (41). Our findings also underline the importance of Nrf2 in response to NiSO_4_ since the Nrf2 stress response signaling was identified as highly activated pathway using IPA analysis. In this context, we were able to identify significantly increased gene expression levels for the Nrf2 target genes peroxiredoxin (PRDX) 1, SOD2, NQO1, glutathione S-transferase (GST) M1 and GSTO1 as well as elevated protein levels of SOD2 and HMOX1. Besides NiSO_4_, LPS is also able to induce oxidative stress in dendritic cells which is believed to support the maturation of the cells (42, 43). We identified SOD2 as strongly upregulated protein after treatment with LPS which was verified by quantitative RT-PCR. Proteomic upregulation of SOD2 after LPS-treatment was also reported by Hussaarts et al. (3). Thus, Nrf2-dependent proteins play a major role during cellular response to an allergen. However, biomarker candidates from this group of proteins have to be selected carefully since other substances like LPS that induce DC maturation also regulate Nrf2-dependent proteins.

The induction of TLR4 signaling pathways by binding of nickel undoubtedly contributed to the activation of the cells as proposed by Schmidt et al. (18). However, Vennegaard et al. (44) showed that sensitization to Ni^2+^ occurs independent of TLR4 in mice. MyD88-dependent and IL-1-related pathways mediated the immunological reaction to Ni^2+^ in their *in vivo* model. In our study with human MoDCs, we found significantly elevated secretion levels of IL-1β after treatment with NiSO_4_, which may be indicative for an involvement of IL-1-related pathways during sensitization to NiSO_4_. We also believe that the haptenation of nickel to other cellular proteins led to the induction of a distinct oxidative stress response which eventually resulted in cellular activation and the observed proteomic phenotype of the MoDCs. The haptenation mechanisms of metal allergens remain to date under-investigated. Yet, nickel was shown to bind to a broad range of cellular proteins, including many heat shock proteins and cytoskeletal proteins (45, 46). Binding of nickel to serum albumin was shown to bypass antigen-processing of antigen-presenting cells by a transfer of the Ni^2+^-ion to peptide-major histocompatibility complexes inducing subsequent T cell activation (45). Due to the *modus operandi* in this study, further nickel-interacting proteins could not be detected.

IPA results revealed the superpathway of cholesterol biosynthesis as strongly induced due to NiSO_4_- but not after LPS-treatment. NiSO_4_-treated MoDCs exhibited significantly reduced cellular cholesterol levels which were accompanied by upregulation of genes and proteins involved in the cholesterol biosynthesis. Cellular cholesterol homeostasis is a delicate equilibrium. Under cholesterol deficiency, sterol response element-binding proteins (SREBP) become activated and subsequently activate genes of the mevalonate pathway like HMGCR to fuel cholesterol accumulation (47). Changes in cholesterol levels can lead to fatal effects with major impacts on DCs role in immunity. Disrupted cholesterol homeostasis may affect DC differentiation and maturation, antigen presentation, migration, as well as priming of T cells (48). During DC maturation cholesterol highly supports the synthesis of membranes needed for cellular expansion. Especially the formation of lipid rafts in the cell membrane is sensitive to changed cholesterol levels. Since TLR or major histocompatibility complexes are located in these rafts, receptor-induced signaling like NFκB- or IFN-signaling is directly affected upon perturbed cholesterol levels (48–51). Excessive cholesterol biosynthesis may also be a cellular mechanism to restore and maintain cell membranes. Cholesterol decreases membrane fluidity and therefore stabilizes the latter (52).

Cholesterol is also known to play a pivotal role in cellular stress response and during hypoxia. Under hypoxic conditions, sterol levels were depleted in the yeast *Saccharomyces pombe*, which was counteracted by activation of Sre1 (the analog of SREBPs in eukaryotes) until normal levels of sterol synthesis were again reached after a few hours (53). In our experiment, the gene expression of SREBP and HMGCR was continuously upregulated in NiSO_4_-treated cells. Thus, potentially being a compensatory effect for the cellular cholesterol depletion induced by NiSO_4_. A recently published study on the lipid composition in maturated human MoDCs, revealed that mature MoDCs are stiffer than their immature progenitors. These findings were explained by an altered lipid class composition in mature MoDCs. Strikingly the performed lipidomic analysis also disclosed reduced cholesterolester levels in mature MoDCs (54), which supports our findings of cellular cholesterol depletion. However, the role and fate of cholesterol during DC maturation is to date not fully understood.

We suspect that diminished cellular cholesterol levels can be explained by the formation and secretion of extracellular vesicles. Extracellular vesicles were proven to play a pivotal role in allergy and immunity in general (55). Exosomes of dendritic cells may transport allergens and thereby activate T cells (56). Another example for the relevance of DC exosomes is their use as vaccine in cancer immunotherapy (57). Taken together, the impact of NiSO_4_ on the cellular fate of cholesterol is an exciting target for future research. Hence, we already conducted a subsequent proteomic experiment to unravel whether the observed changes in cholesterol biosynthesis are unique for NiSO_4_. Appling a stable isotope labeling with amino acids in cell culture (SILAC), we analyzed the effects of organic allergens, including the strong allergens *p*-benzoquinone and 2,4-dinitrochlorobenzene, on the proteome of THP-1 cells. THP-1 cells are validated for usage within the human Cell Line Activation Test to assess skin sensitizers (26). We were able to confirm the upregulation of pathways connected to the cholesterol biosynthesis after treatment of THP-1 cells with all contact sensitizers tested (unpublished data). These data support the evidence presented in our study in MoDCs and underline the need for further research in this field which may eventually lead to a better understanding of cellular mechanisms in the context of ACD.

Due to strict search criteria for the IPA pathway analysis, no metabolic pathways were identified as significantly regulated. However, as already described in the literature, phenotypic alterations and cellular reconstruction during DC maturation are supported by a metabolic reprogramming, i.e. cellular metabolism is shifted from oxidative phosphorylation toward glycolysis which fuels the cells with sufficient energy. The metabolic shift toward glycolysis is triggered by strong and weak DC activators likewise and is essential for DC migration (58). However, long-term dependency on glycolysis was only induced by potent DC activators and is achieved by HIF1α stabilization (59, 60). As reported before, LPS triggered a metabolic shift toward glycolysis by increasing the gene expression of HK2 (30). Although protein levels of HK2 were not significantly changed due to LPS, HK2 was one of the proteins with the most pronounced differential expressions after NiSO_4_-treatment. HK2 protein expression was significantly altered and 3.4-fold upregulated, respectively. Additionally, we identified another protein supporting the shift toward glycolysis: PFKFB3 was significantly upregulated after LPS- and NiSO_4_-treatment. PFKFB3 is a kinase producing fructose 2,6-bisphosphate which is an allosteric activator of the phosphofructokinase, a bottleneck enzyme in the glycolysis. PFKFB3 was reported to increase glycolytic activity in cells in the context of the Warburg effect (61) as well as after LPS stimulation of macrophages (60). As the PFKFB3 gene promotor possesses a binding site for HIF1α (62), it is also induced under hypoxic and hypoxia-like conditions, e.g. triggered by Co^2+^ (63).

The findings, as mentioned above, can be matched to the proteomic data generated from MoDCs treated with NiSO_4_. However, NiCl_2_ was reported to induce distinct hypoxia-like conditions via induction of HIF1α (64, 65). Accordingly, increased gene expression for glycolytic enzymes like HK2, LDHA and SLC2A1 was reported (66). These proteins were also significantly changed after treatment of MoDCs with NiSO_4_ in our study, and additionally, elevated protein levels of HIF1α were detected. MoDCs stimulated with NiSO_4_ were more sensitive to HIF1α-inhibition than cells stimulated with LPS. NiSO_4_ and cobalt are known to induce TLR4-dependent IL-8 production, which is defined as a key event for skin sensitization. However, both metals also elicit hypoxia. Asakawa et al. (67) showed that NiCl_2_ binds to heat shock protein 90β in THP-1 cells and thus increases the activity of HIF1α. Elevated HIF1α activity eventually resulted in elevated IL-8 expression. TREM1 protein expression and IL-8 secretion were both previously reported to be increased under hypoxic conditions (68). We could show that NiSO_4_-treated MoDCs also induced TREM1 signaling as well as IL-8 secretion. Additionally, Viemann et al. (65) described a central role of HIF1α in endothelia cells in response to nickel. Besides pro-inflammatory signaling by NFκB, HIF1α was identified as a central inducer of genes that act on cell survival and metabolism. Hypoxia and HIF1α were found to be elementary drivers during DC activation and thereby enhancing the immunological response of the latter. Precisely, hypoxia was shown to enhance pro-inflammatory signaling, cellular glucose metabolism as well as cell surface maker expression in mouse-derived DCs. This eventually resulted in an amplified ability to stimulate T cell proliferation (59). We could confirm the metabolic shift in NiSO_4_-treated cells along with the upregulation of important cell surface makers and the ability to stimulate lymphocyte proliferation. Hence, the induction of a pronounced hypoxia by NiSO_4_ contributed strongly to the immunological response of the MoDCs. However, more data is needed to allow distinct discrimination between TLR4-dependent and hypoxia-induced proteins in NiSO_4_-treated MoDCs. Furthermore, the pronounced Nrf2-mediated stress response may also favorably contribute to HIF1α-dependency in NiSO_4_-treated MoDCs since Nrf2 directly regulates HIF1α gene expression (69).

## Conclusion

In the present work, we extensively studied the activation of MoDCs by mass spectrometry-based proteomics. We aimed to elucidate the cellular mode of action of NiSO_4_-induced MoDC maturation concerning nickel allergy by comparing MoDC activation induced by NiSO_4_ to the well-studied bacterial LPS. Overall, the results for LPS-treated MoDCs are in concordance with literature data, confirming that our technical approach detects DC activation as such. NiSO_4_-treated cells upregulated relevant activation markers, such as CD83 and CD86, and pathways that point toward signaling in immunology, like CD40 and TREM1 signaling. However, the immunological response triggered by NiSO_4_ is partly overshadowed by oxidative stress that is potentially caused by the metallic character of Ni^2+^. This implicates a subordinate role of TLR4-activation and -signaling in NiSO_4_-activated MoDCs for the chosen experimental settings. NiSO_4_-induced oxidative stress is likely the responsible event upstream of the upregulation of Nrf2-target genes, as detected at mRNA as well as at protein level. The metabolic shift observed for NiSO_4_-treated MoDCs resembles that of LPS-treated MoDCs. Nevertheless, prolongation of the upregulation of detected glycolytic enzymes, in connection with elevated HIF1α levels, suggest a hypoxia-like cellular state, which is remarkably different from LPS-induced effects. The link between cellular cholesterol depletion and activation of DCs triggered by NiSO_4_ remains to be elucidated by future studies.

Taken together, our study suggests immunostimulatory and cytotoxic effects of NiSO_4_ leading to DC activation, and possibly culminating in sensitization to nickel *in vivo*. Our results illuminate the phenotype of activated DCs during sensitization to an allergen and thereby enhance our understanding of nickel-induced molecular pathways.

## Data Availability Statement

The dataset presented in this study can be found online in the PRIDE repository with the dataset identifier PXD022599 (70). A full list of all proteins including the protein names and accession numbers can be found in the article/Supplementary Material.

## Ethics Statement

The studies involving human participants were reviewed and approved by Charité, Berlin Germany; EA4/071/13. Written informed consent for participation was not required for this study in accordance with the national legislation and the institutional requirements.

## Author Contributions

TH and AH conceived and designed the experiments. TH performed the experimental work. Data analysis was done by TH, VD, and KSc. Figures were created by TH and KSc. KSi and TH conceived, conducted, and analyzed the mixed leukocyte reaction. MB provided conceptual input. The manuscript was written by TH, KSc, and AH with contributions from all co-authors. All authors contributed to the article and approved the submitted version.

## Conflict of Interest

The authors declare that the research was conducted in the absence of any commercial or financial relationships that could be construed as a potential conflict of interest.

## Abbreviations

ACD: allergic contact dermatitis
ACN: acetonitrile
ALDOA: aldolase A
APC: allophycocyanin
BV421: brilliant violet 421
CD: cluster of differentiation
CFSE: carboxyfluorescein succinimidyl ester
CYP51A1: lanosterol 14α-demethylase
DC: dendritic cell
EIF: eukaryotic initiation factor
FA: formic acid
FC: fold change
FITC: fluorescein isothiocyanate
GLUT: glucose transporter type
GM-CSF: granulocyte-macrophage colony-stimulating factor
GST: glutathione S-transferase
HEPES: 4-(2-hydroxyethyl)-1-piperazineethanesulfonic acid
HIF: hypoxia-inducible factor
HK: hexokinase
HMGCS1: hydroxymethylglutaryl-CoA synthase
HMGCR: 3-hydroxy-3-methyl-glutaryl-coenzyme A reductase
HMOX: heme oxygenase
HPRT: hypoxanthineguanine phosphoribosyltransferase
HSA: human serum albumin
IL: interleukin
IPA: ingenuity pathway analysis
KEAP: kelch-like ECH-associated protein
LDHA: lactate dehydrogenase
LFQ: label-free quantification
LPS: lipopolysaccharide
MD-2: myeloid differentiation factor 2
MLR: mixed leukocyte reaction
MoDC: monocyte-derived dendritic cell
NEAA: non-essential amino acids
NFκB: nuclear factor κB
NQO: NAD(P)H dehydrogenase [quinone]
Nrf2: nuclear factor erythroid 2-related factor 2
OECD: Organization for Economic Co-operation and Development
PBMC: peripheral blood mononuclear cell
PBS: phosphate buffered saline
PE: phycoerythrin
PFKFB: 6-phosphofructo-2-kinase/fructose-2,6-biphosphatase
PRDX: peroxiredoxin
q(RT)-PCR: quantitative real time polymerase chain reaction
SD: standard deviation
SLC: solute carrier
SOD: superoxide dismutase
SREBP: sterol regulatory element-binding protein
TLR: toll-like receptor
TNBS: trinitrobenzenesulfonic acid
TNF: tumor necrosis factor
TNP: trinitrophenyl-
TRAF: TNF receptor-associated factor
TREM: triggering receptor expressed on myeloid cells.
