# Supplementary material for: The Contact Allergen NiSO4 Triggers a Distinct Molecular Response in Primary Human Dendritic Cells Compared to Bacterial LPS
Source: Front Immunol. 2021 Mar 11;12:644700. doi: 10.3389/fimmu.2021.644700 (PMC7991087; doi:10.3389/fimmu.2021.644700)
Supplement: Supplementary file 1 [file Presentation_1.pdf]

Supplementary Material

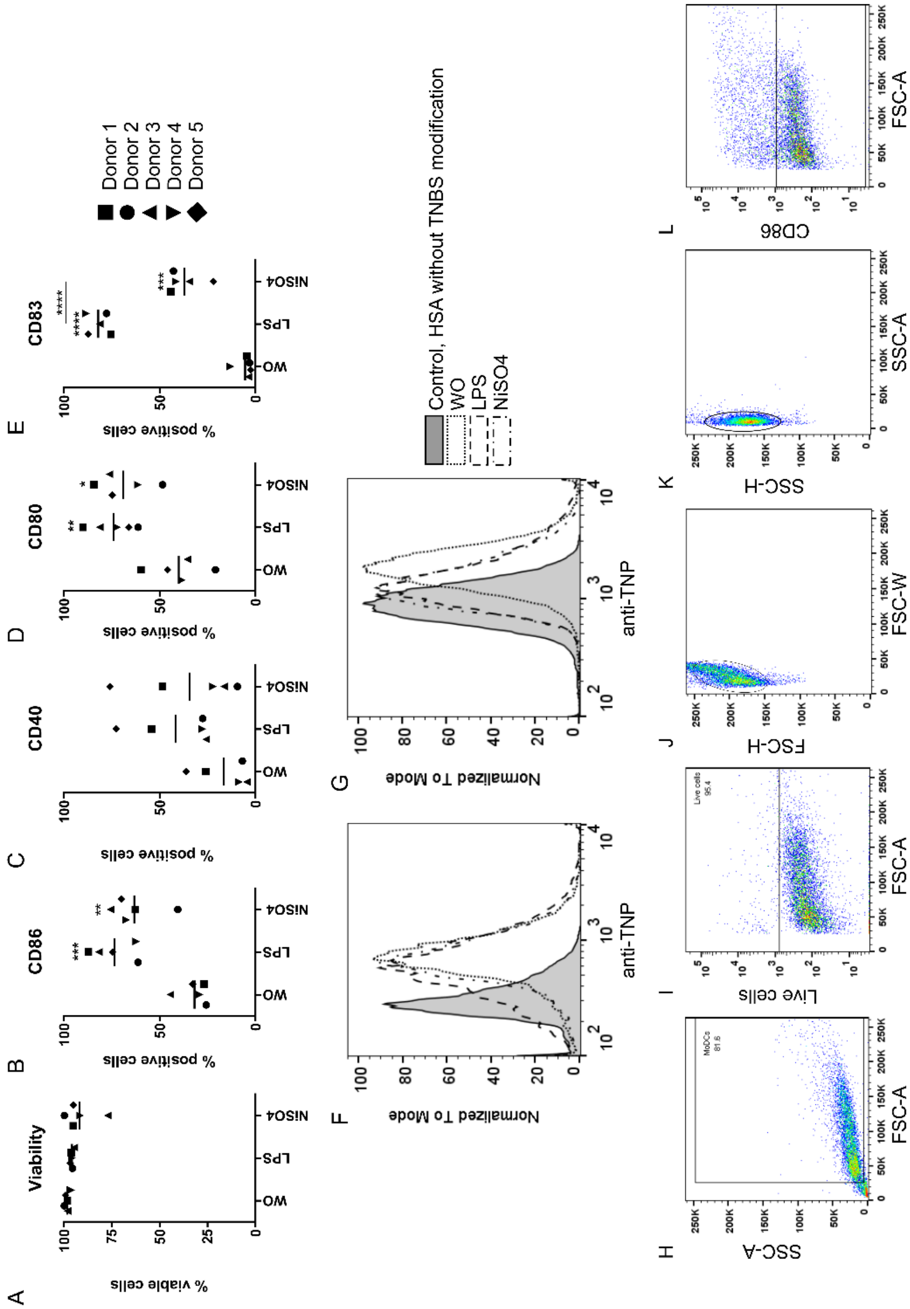

Supplementary Figure 1: Surface marker expression and protein uptake of LPS- and $NiSO_4$-treated MoDCs. Immature MoDCs were treated for 24 h with 400 µM $NiSO_4$ or 2.5 µg/ml LPS. (A) Cell viability was assessed by flow cytometry using near-IR dead cell stain. Induced expression of the activation markers CD86 (B), CD40 (C), CD80 (D), CD83 (E) on the live cells depicted in (A).

Depicted are the means as well as individual percent values of live cells or positive cells, respectivley (n = 5 donors). Significance to medium control was calculated using Student's t-test (*: p-value ≤ 0.05; **: p-value ≤ 0.01; ***: p-value ≤ 0.001). (F, G) Phagocytic activity was assessed by HSA-TNP uptake of MoDCs treated with $NiSO_4$ or LPS for 3 h (F) or 24 h (G). One representative experiment out of three is shown. (H-L) Gating strategy for MoDCs: Cells were gated based on size (H), live cells (I), FSC (J) and SSC (K) single cells and the marker of interest (L).

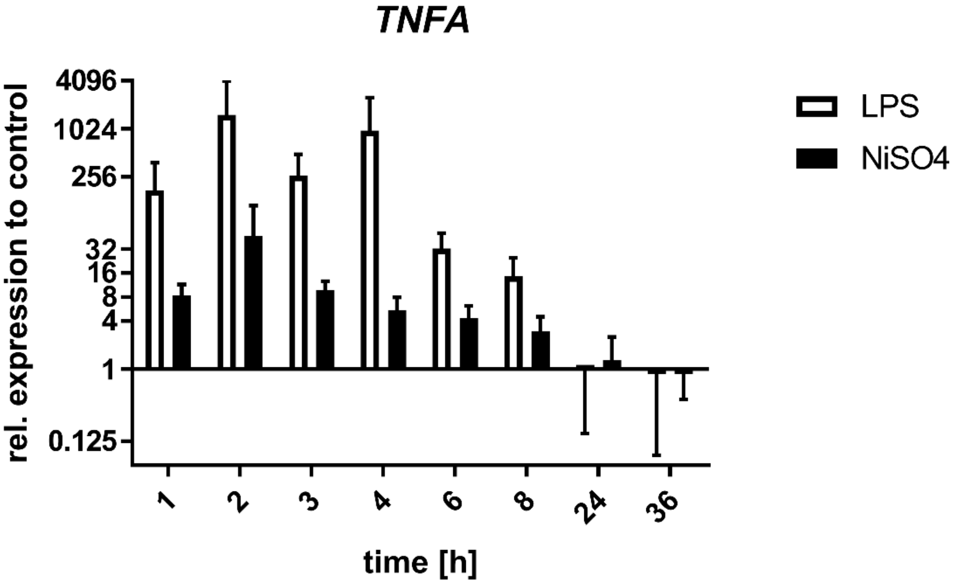

Supplementary Figure 2: Relative TNFA gene expression after treatment with 2.5 µg/ml LPS or 400 µM $NiSO_4$ at the indicated time points. Gene expression was normalized to the gene expression of HPRT (housekeeping gene) and medium control. Means and SDs of the normalized data are shown (n = 3 donors).

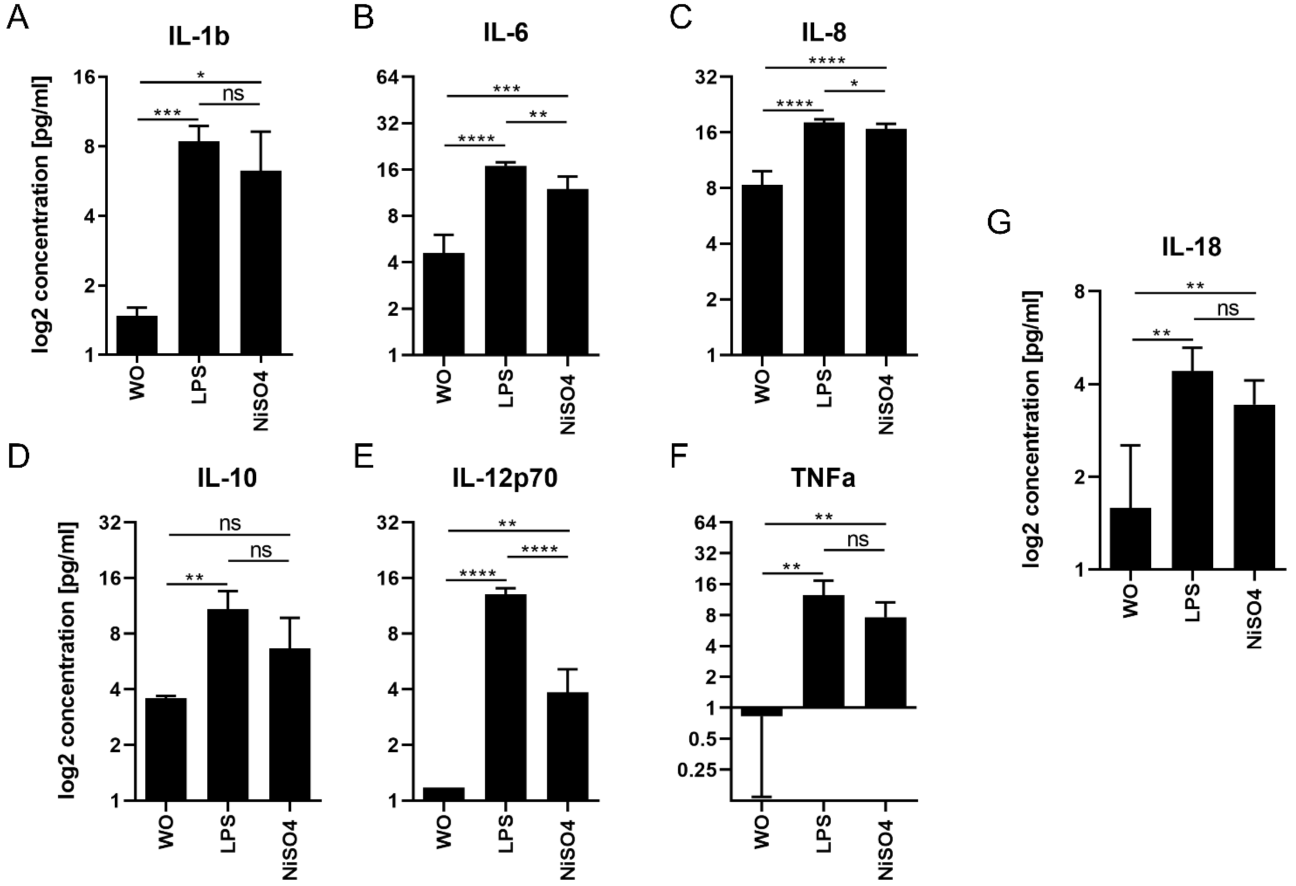

Supplementary Figure 3: Quantification of secreted cytokines in the cell culture supernatant after 24 h treatment of MoDCs with 400 µM $NiSO_4$, 2,5 µg/ml LPS or medium only (WO). IL-1β, IL-6 and IL-8, IL10, IL12p70, IL-18 and TNFα were quantified using a bead-based assay. Shown are means and SDs of the log2 concentrations (n = 5 donors). Significance to medium control was calculated using Student's t-test (ns: not significant; *: p-value ≤ 0.05; **: p-value ≤ 0.01; ***: p-value ≤ 0.001; ****: p-value < 0.0001).

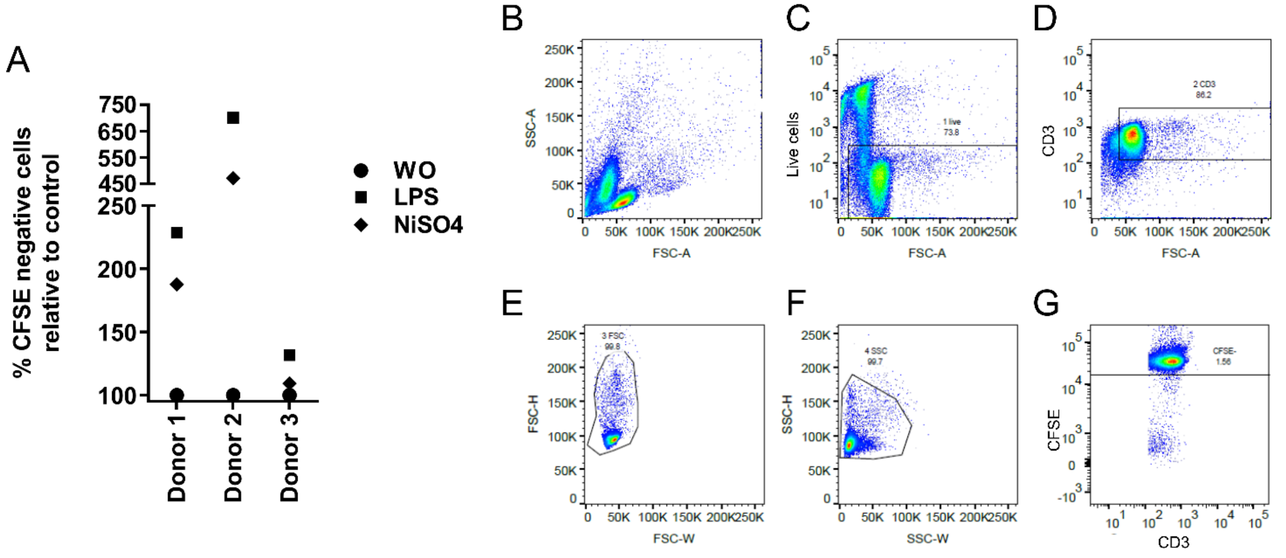

Supplementary Figure 4: T cell proliferation was assessed in a mixed leukocyte reaction via CFSE dilution. (A) % CFSE negative cells on day 4 of the mixed leukocyte reaction relative to medium control (set to 100 %). Allogenic T cells were stimulated by MoDCs treated for 24 h with 2.5 µg/ml LPS, 400 µM $NiSO_4$ or medium only (n = 3 donor pairs). (B-G) Dot plots of CFSE-negative and $CD3^+$ T cells on day four of the mixed leukocyte reaction. Cells were gated for live cells (C), $CD3^+$ cells (D), FSC (E) and SSC single cell gate (F) and CFSE (G).

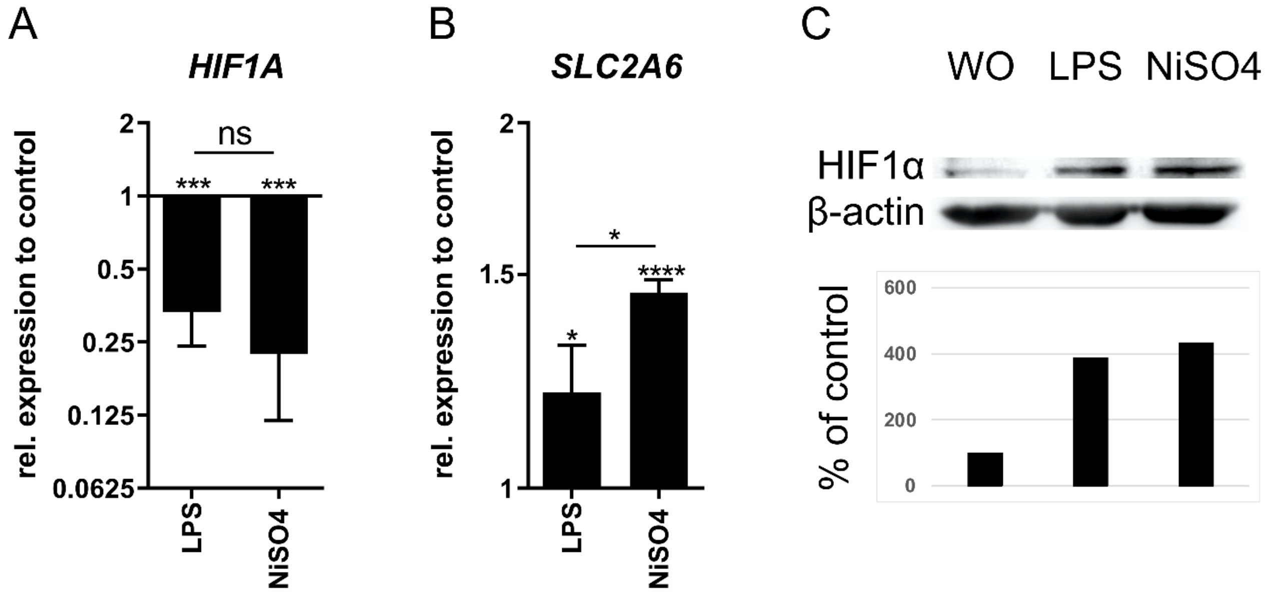

Supplementary Figure 5: Relative gene expression as well as protein levels of proteins involved in glucose metabolism. Relative gene expression of hypoxia-inducible factor 1-α (HIF1α, A) and solute carrier family 2 member 6 (SLC2A6, B). Gene expression was measured by qRT-PCR and normalized to the gene expression of HPRT (housekeeping gene) and medium control. Means and SDs of the normalized data are depicted (n = 3 donors). MoDCs were treated with medium only, 2.5 µg/ml LPS or 400 µM $NiSO_4$ for 24 hours. Significance to medium control was calculated using Student's t-test (ns: not significant; *: p-value ≤ 0.05; **: p-value ≤ 0.01; ***: p-value ≤ 0.001; ****: p-value < 0.0001). (C) HIF1α immunoblot of LPS- and $NiSO_4$-treated MoDCs. Cells were harvested after 24 h of treatment. HIF1α was quantified by standardizing the specific band to the respective β-actin loading control. These ratios were normalized to medium only as control (WO). One representative immunoblot is shown (n = 3 donors).

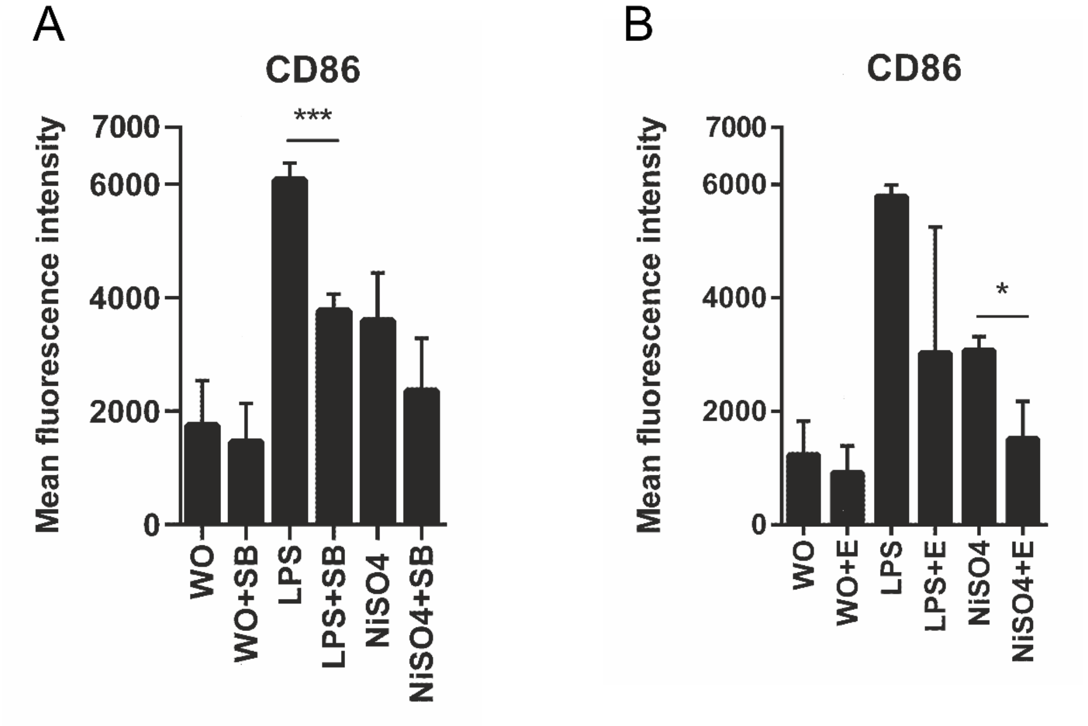

Supplementary Figure 6: Effects of p38 inhibition (A) and HIF1α inhibition (B) on the mean fluorescence intensity of CD86. 10^5 cells were incubated with 20 µM SB203580 or 0.5 nM echinomycin and medium only as control (WO). After 30 min, cells were additionally stimulated with medium only, 2.5 µg/ml LPS or 400 µM $NiSO_4$ for 24 h. CD86 expression was monitored by flow cytometry. Means and SDs are plotted (n = 3 donors). Significance was calculated using Student's t-test with *: $p \leq 0.05$; **: p-value ≤ 0.01 and ***: p-value ≤ 0.001.
